# Female residents experiencing medical errors in general internal medicine: a qualitative study

**DOI:** 10.1186/1472-6920-14-140

**Published:** 2014-07-10

**Authors:** Cindy Ottiger Mankaka, Gérard Waeber, David Gachoud

**Affiliations:** 1 Medical School, Faculty of Biology and Medicine, University of Lausanne, 1011 Lausanne, Switzerland; 2 Service of Internal Medicine, University Hospital of Lausanne (CHUV), 1011 Lausanne, Switzerland; 3 Educational Unit, Faculty of Biology and Medicine, University of Lausanne, Rue du Bugnon 21, BU21/05-246, 1011 Lausanne, Switzerland

**Keywords:** Medical errors, Patient safety, Medical education, Internal medicine, Gender, Qualitative research

## Abstract

**Background:**

Doctors, especially doctors-in-training such as residents, make errors. They have to face the consequences even though today’s approach to errors emphasizes systemic factors. Doctors’ individual characteristics play a role in how medical errors are experienced and dealt with. The role of gender has previously been examined in a few quantitative studies that have yielded conflicting results. In the present study, we sought to qualitatively explore the experience of female residents with respect to medical errors. In particular, we explored the coping mechanisms displayed after an error. This study took place in the internal medicine department of a Swiss university hospital.

**Methods:**

Within a phenomenological framework, semi-structured interviews were conducted with eight female residents in general internal medicine. All interviews were audiotaped, fully transcribed, and thereafter analyzed.

**Results:**

Seven main themes emerged from the interviews: (1) A perception that there is an insufficient culture of safety and error; (2) The perceived main causes of errors, which included fatigue, work overload, inadequate level of competences in relation to assigned tasks, and dysfunctional communication; (3) Negative feelings in response to errors, which included different forms of psychological distress; (4) Variable attitudes of the hierarchy toward residents involved in an error; (5) Talking about the error, as the core coping mechanism; (6) Defensive and constructive attitudes toward one’s own errors; and (7) Gender-specific experiences in relation to errors. Such experiences consisted in (a) perceptions that male residents were more confident and therefore less affected by errors than their female counterparts and (b) perceptions that sexist attitudes among male supervisors can occur and worsen an already painful experience.

**Conclusions:**

This study offers an in-depth account of how female residents *specifically* experience and cope with medical errors. Our interviews with female residents convey the sense that gender possibly influences the experience with errors, including the kind of coping mechanisms displayed. However, we acknowledge that the lack of a direct comparison between female and male participants represents a limitation while aiming to explore the role of gender.

## Background

Facing medical errors is unfortunately a common experience for doctors-in-training such as residents. By definition, they are at higher risk of committing errors during the stage of first developing medical competences [1,2]. Residents deserve particular attention because behaviours learnt early in the practice are likely to persist later in professional life [3,4]. A resident’s experience with an error is shaped by three primary elements: (a) the individual profile of the resident, (b) the nature of the error, and (c) the context in which the resident works [4]. The last two elements have been explored in studies on residents’ experiences with errors [1-8], whereas the first element - the individual profile of the resident - has been much less explored. However, a comprehensive approach to errors during residency should include the role played by the residents’ individual profile.

The few data on doctors’ individual profile come mainly from surveys. In several of those [2,5,8-10], the issue of gender was analyzed. According to these surveys, the doctors’ gender appeared to influence the experience with errors in the three following areas: (a) self-reporting of errors, (b) reactions to errors, and (c) changes undertaken in the practice after an error. Some of the published findings on gender effects are conflicting, as described below.

### Self-reporting of errors

In one study that included 184 participants, the rate of self-reported errors did not vary significantly between female and male residents [5].

In contrast, Aasland [10] found a gender difference where being a male doctor was a predictor for increased self-reporting of errors (OR = 1.97; 95% CI 1.43 ‾ 2.90).

### Reactions to errors

In a survey of 3’171 physicians practicing in Canada and the US [9], female physicians were almost twice as likely to report stress after an error than their male counterparts (OR = 1.91; 95% CI = 1.21 ‾ 3.05).

### Changes undertaken after an error

In a survey of 254 internal medicine house officers [2], Wu conducted a multivariate analysis and found that female gender was significantly associated with undertaking constructive changes in the practice.

Considering these studies with their quantitative approach to a complex problem, we sought to gather in-depth data from our own working establishment to better understand the role of gender. There is no doubt that the net effect of gender would be best analyzed by comparing both female and male residents’ experiences with errors. Nevertheless, we decided to focus on thorough accounts of *female* residents since those were missing from the public domain. Collating these accounts would give a more accurate picture of whether there are commonalities among female residents’ experiences with medical errors.

In relation to the role of gender, it is useful to provide some background information about the national context of this study. It was conducted in Switzerland, where the medical profession has feminized in similar proportions to other Western countries [11]. Whereas 60% of Swiss medical graduates are female, career development is still substantially different between female and male doctors in Switzerland. This has been recently shown by Buddeberg in the SwissMedCareer Study [11]. In this study, she noticed a gender-based segregation at both a horizontal and vertical level. The horizontal segregation locates female doctors in less recognized medical specialties, while the vertical one locates them at a lower stage in the academic and hospital hierarchy.

Experiencing medical errors leads to engage in various coping mechanisms [12]. Indeed, medical errors can have a severe psychological impact on doctors, who have been described as the second victims [13]. Whatever the severity of psychological stress, humans manage the situation by engaging in coping mechanisms [12]. These mechanisms include all the cognitive and behavioural efforts needed to manage stressful demands exceeding one’s own current resources [14]. Coping mechanisms are of paramount importance in the error experience because they are intrinsically bound to the changes an individual may - or may not - undertake after an error [2,13]. For example, coping by denying personal responsibility limits one’s own involvement in improving the system.

On this background we aimed to answer the following two research questions:

1) How do female residents experience medical errors?

2) Which coping mechanisms do female residents use?

## Methods

Because we were interested in gathering in-depth data and because individual experiences were the focus of this study, we chose a qualitative approach called phenomenology. In this approach, the researcher explores the experience of the participants, as they both lived and gave meaning to the said experience [15,16].

According to phenomenology, we used interviews because they generate understanding of the meanings different individuals have of their lived world [17]. A questionnaire was developed to conduct the interviews, which were thereby semi-structured. It entailed a set of open, pre-defined questions. Table‒ 1 shows the questions asked to the participants.

**Table 1:** Questions asked during the interviews

**Domains covered**	**Questions asked**
General considerations about medical errors	1. How would you define a medical error?
Residents’ personal experiences with errors	2. Did you experience a medical error?
	Could you describe this experience and explain how you were involved?
	3. If you think again about that time, do you remember how you felt?
	4. How did your supervisors, colleagues and relatives react at that time?
	5. Did you feel supported by your supervisors and colleagues?
	6. Which were the main factors that contributed to this error?
	7. Today, how do you feel about this error?
	8. How did this error impact on your private and professional life?
	Were they long-term consequences?
Residents’ ways of coping	9. If you think again about that time, which resources did you use to manage the error?
	10. Could you have used other resources?
Gender issues and errors	11. Do you think that being a woman could have influenced your experience or management of this error?
	Could you provide an example?

### Setting and sample

We used purposeful sampling technique. In the qualitative paradigm, this basically means identifying individuals who are able to provide the researchers with information-rich accounts about the question under study [18]. In regard to our research questions, we opted for typical cases of female residents having experienced a medical error and willing to share their story.

We limited our sampling to residents in only one medical specialty: general internal medicine. This focus on a single specialty has the advantage of reducing the variability of experiences and having thereby a more homogenous sample. In addition, general internal medicine in our context is a core discipline that many doctors-in-training accomplish as part of postgraduate education. The study took place in the department of internal medicine of a Swiss university hospital.

### Recruitment and data collection

A total of 57 female residents were working in general internal medicine when the recruitment started. Successive series of 5 to 10 emails were sent to our target population. Eventually, 35 recruitment emails were sent, which allowed us to recruit and interview eight female residents. Because recruitment and interviews ran in parallel with data analysis, we included the number of residents that would let us reach thematic saturation [18].

The first author (COM) conducted all the interviews, outside of the current workplace of the participants (i.e. in another building). Residents’ names were only known to COM, who was a final-year medical student at the time of the study and had no work relationship with the participants. Because the interviewer’s professional experience was less advanced than interviewees’ one, a nonjudgmental climate prevailed during the interviews, which favored an open discussion about medical errors. This was eventually confirmed by a number of poignant and emotional testimonies reported by the participants. To favor an open discussion about the role of gender, the interviewer was female, like the participants.

Participants were between 28 to 33 years old and within their second to sixth years of postgraduate training. All the participants displayed the common characteristic of desiring to share information about errors for which they had some level of responsibility. This required from them to have enough insight to recognize such situations.

According to the data and the themes emerging from them, the participants appeared to have one main reason for taking part in the interviews: the genuine sense that reporting their own experience will contribute to shed more light on the problem of medical errors. Some participants certainly hoped that their interviews could contribute to change a system in which they had suffered as second victims [13].

Interviews lasted between 30 minutes to one hour. These were audio-recorded and transcribed verbatim for analysis [18]. Anonymity was guaranteed to all the study participants. Any identifier - such as names of persons or hospitals - was removed from the interview transcripts. For ease of handling, all data were input into a PC word processing package, which gave a good degree of flexibility to manipulate the data.

### Data analysis

Before describing how practically the data were analyzed, this section summarizes the authors’ background to better locate their standpoint in the analysis process. All three authors belong to the medical profession: COM is a final-year medical student, whereas GW and DG are clinicians with long experience in general internal medicine. All three authors share a common interest for the theme of medical errors: COM anticipates the future challenges of her profession. GW and DG aim at reducing the burden of errors on the patients they care for and on the trainees they supervise.

With regard to the process of analyzing the data, the interview transcripts first underwent an inductive thematic analysis to allow emergence of the meanings the participants gave to their lived experience with medical errors. Although the analysis was mainly inductive, a deductive component still played a role in the process since our questionnaire gave a general structure to the interview and defined the domains to explore. The content of the transcripts was fragmented in multiple categories. Each of these categories was made of a set of related quotes reflecting a similar idea. This first phase - performed by COM - allowed generation of an initial framework of categories. The last author (DG) checked all the categories and all the quotes extracted from the eight transcripts. Any disagreement on the categories was resolved through discussion until consensus was reached. In a second phase, the framework was refined to organize all the previously identified categories into a set of final themes and subthemes; the “comparative method” was also applied [19]. This involved comparing all the data belonging to the same theme and subtheme to ensure there was appropriate conceptual similarity.

### Ethics

The study was approved by the local ethics board, the Human Research Ethics Committee of Vaud. Participants were asked to sign an informed consent form at the beginning of the interview.

## Results

The errors reported by the participants are described in Table‒ 2, which also includes the hospital sites where they occurred.

**Table 2:** Description of reported errors with patient outcomes and hospital types

**Errors’ description with patient outcomes and an example**	**Number of errors**
Missed diagnosis and/or inadequate surveillance, resulting in patient death	2
*Example: Early signs of septic shock in a patient with cirrhosis were not identified*	
Missed diagnosis and delayed treatment, resulting in possible long term consequences for the patient	1
*Delayed treatment of an acute coronary syndrome*	
Missed diagnosis, resulting in patient readmission	1
*Missed diagnosis of pulmonary embolism during emergency outpatient visit*	
Missed diagnosis and/or delayed treatment, without any long-term consequences	4
*Diabetic ketoacidosis misdiagnosed as a hyperventilation crisis*	
Medication error, resulting in severe but completely reversible consequences	1
*Error in prescription of opiates resulting in respiratory failure*	
**Hospital sites where the errors occurred**	**Number of errors**
Community hospitals in Switzerland	6
(including ER and ICU where residents in internal medicine rotate)	
University hospitals in Switzerland	2
Other hospitals (outside of Switzerland)	1

The analysis generated seven main themes, with their related subthemes. They are shown in Table‒ 3. We illustrate in the same table the relationship between the domains explored during the interviews (first column) and the themes that emerged from the analysis (second column).

**Table 3:** Themes and subthemes, in relation to the interview’s structure

**Domains explored during the interviews**	**Themes**	**Subthemes**
General considerations about medical errors	1. Insufficient culture of safety and error	-
Residents’ personal experiences with errors	2. Perceived causes of errors	Fatigue
		Stress and work overload
		Inadequate level of competences in relation to assigned tasks and/or
		inadequate supervision
		Dysfunctional communication
	3. Negative feelings in response to errors	Emotional distress
		Guilt/self-blame
		Self-doubt/loss of confidence
		Anger against self
	4. Variable attitudes of the hierarchy	From very supportive to not supportive at all
Residents’ ways of coping	5. Talking about the error	Talk to family members
	‾ as the core coping mechanism	To friends
		To peers and colleagues
		To supervisors
		Disclose the error to the patient
	6. Defensive and constructive attitudes towards errors	Share responsibility of the error with others
		Blame the system
		Consider the problem of error as normal
		Learn from one’s own errors and make changes in the future
Gender issues and errors	7. Gender-specific experiences in relation to errors	Male residents perceived as more confident and less affected by errors
		Perceptions that sexist attitudes among male supervisors can occur

In the following sections, each theme is discussed with relevant quotes. The first name indicated after each quote is an alias.

### Insufficient culture of safety and error

A recurrent issue in participants’ statements is the lack of a culture conducive to addressing errors openly and constructively. Too often, the residents did not feel comfortable to speak of their errors within their work environment, for fear of being either blamed or stigmatized as weak.

*“In the medical environment, there is no culture of feedback, but [there is] a competitive atmosphere where showing any sign of weakness makes you become the weak link⋯”* - Vanessa

### Perceived main causes of errors

Among the perceived causes of errors, three were predominant in participants’ accounts: (a) stress and time pressure linked to work overload, (b) fatigue, and (c) inadequate supervision or inadequate level of clinical competence in relation to the tasks.

The following resident discussed the level of supervision provided in smaller hospitals:

*“In smaller hospitals, there are fewer staff members, and [as residents] we feel lonelier because there is less supervision. Sometimes, there is only one attending above us, and no chief resident. When we have a question, it has to be carefully selected because the supervisor is often very busy.” -* Andrea

The following resident commented on an error involving a patient with a status epilepticus:

*“We don’t have enough experience to take care of such a situation. We know a lot of theoretical things, but the whole medical practice has to be learnt⋯ The responsibilities we have as young residents are huge and out of proportion to the training we received. It is frightening⋯” -* Clothilde

### Negative feelings in response to errors

In this theme are included all kinds of negative feelings that the participants experienced after the error. These negative feelings can be classified in four subthemes: (a) *emotional distress* that manifested in psychological reactions characterized by suffering, such as crying or feeling psychologically unwell, (b) *guilt* that the participants felt when blaming themselves for the error (c) *self-doubt* when they called their own competences into questions, and (d) *anger* that a few participants experienced, as a reaction directed against themselves*.*

The following excerpts exemplify these negative feelings. The first resident commented on the case of a cirrhotic patient, whose early signs of septic shock were missed. The patient died the same night.

*“We are here [as doctors] to save people, not to kill them [⋯]. I was feeling extremely bad, like a dead loss, guilty, dangerous [⋯]. Then, I had to go on and see new patients. It was hard⋯ I was so frightened to see new patients, even for a rhinitis. This very day, I tried to see as few patients as possible and, between each patient, I was going to the toilets to cry [in secret].”* - Magali

This second resident explained how she had doubts about her competences:

*“I said to myself I have perhaps no chance to become a good doctor [⋯]. Two weeks after the event, I was going to resign: I went to my supervisor’s office, but he wasn’t here. [Retrospectively, I think] I was lucky that he wasn’t here.”* ‾ Sara (laughing at the end)

### Variable attitudes of the hierarchy

In this theme, participants described how they had been supported by their supervisors. Hierarchical support was very diverse across participants’ accounts. Although there were many examples of full support by the hierarchy, a few participants described a total lack of support. The following quotes illustrate this contrast.

*“The day after [the error occurred], I was back to work, and went immediately to the attending. I told him I made a serious error, and I wanted to talk about it. He reacted very well, he listened to me, and we talked together⋯ Then, we went to the ward to see how the patient was doing, to apologize, and to explain to him what had occurred. His support helped me so much⋯” -* Isabelle

In the next example, the participant described the support - or rather the lack thereof - she received after an error leading to the patient’s death (from a shock).

*“The head told me what had happened (i.e. that the patient had died), without any tact. Then, she showed me a chart portraying the physiology of the various kinds of shocks. I couldn’t resist, and I started crying. I asked whether I was dangerous, but she didn’t answer me. I told her I should perhaps stop this job and find another career. At that point, she told me: yes, maybe. I nearly did it⋯”* - Magali

### Talking about the error

As their key coping mechanism, the residents mentioned the need to talk about it. The persons they talk to were diverse: they were the participants’ partners or family members, friends, colleagues, and supervisors as well. Most of the participants were able to find a person they felt comfortable to talk to.

*“My family [members] can’t stand it anymore (laugh): all of them work in health care; we can talk a lot together. They understand. I have also friends who are doctors⋯ I am lucky [because] I can really talk to my relatives and friends. I have a lot of support in my private life.” -* Merry

Surprisingly, *only one* resident reported to have talked to the patient about the error. She found it helpful since disclosing the error was for her an effective way of coping. The other residents gave various explanations for not disclosing the errors. For example, this was the case when the patient had already been transferred to another hospital. An additional explanation for not disclosing the error related to situations in which the error did not have consequences for the patient.

### Defensive and constructive attitudes towards errors

In addition to talking about their error, the participants displayed two types of attitudes towards their errors. On the one hand, there were favorable - or *constructive*^a^*-* attitudes: they include all the actions undertaken to avoid the error is repeated in the future. In our participants’ accounts, the most important attitude of this type was the need to learn from the error. On the other hand, there were unfavorable - or *defensive -* attitudes, which include the efforts made to mitigate one’s own personal responsibility. For example, some residents in our sample were trying to share the responsibility of the error with others.

Both types of attitudes can be seen as ways to cope with the error. They are not mutually exclusive, since some participants were using both in front of the same error.

*“Today, I feel better, but I think I will never forget [this error], and I don’t want to forget because I don’t want that to happen again.” ‾* Clothilde (constructive attitude to avoid repeating the error)

The same resident added:

*“In fact, it is the whole situation that created the problem, and it is not only me and my intervention that made the patient die. This error should be shared [among all the care providers], but they let me carry the can.”* (defensive attitude)

### Gender-specific experiences in relation to errors

During the interviews, the participants were specifically asked to comment on a possible link between gender and their experience with errors (see question 11; Table‒ 1). In response to our question, the participants gave two kinds of accounts.

First, several participants reported their own perceptions of how female and male doctors differ when facing an error. One of these perceptions consisted in male doctors being seen as less sensitive and more confident. This was considered as an advantage since medical culture tends to value and reward self-confidence. According to these participants, male doctors were also perceived as less affected by errors.

The second kind of account was based on personal experiences of perceived sexism. Such accounts were reported by two participants. The accounts of sexism were related to the ways in which male supervisors interacted with female residents involved in an error.

*“It’s true that, when you make a mistake, you’re telling yourself that they [the male supervisors] think you’re the featherbrain of the service. In their opinions, it was almost normal that it was a woman who had made the error. When you are a woman and have the reputation of being a bad doctor, it’s hard to change it, precisely because you’re a woman⋯”* ‾ Magali

Finally, there were other gender issues that the residents mentioned. However, these issues were related to aspects of professional life distinct from errors and were therefore out of the scope of the present study (e.g. female doctors with fewer opportunities for career advancement).

## Discussion

First, our data illustrate that there is still a long way to go before the medical profession can openly and constructively address the problem of errors. Indeed, our participants were almost unanimously complaining about overt deficiencies in this domain. Such statements indicate that medicine, as a profession, has to pursue its efforts to make significant and sustained changes to how it manages the huge problem of medical errors. A thorough journalistic investigation held in 2009 concluded that the death toll due to medical errors had not decreased since the US report “To err is human” published in 1999 [20,21]. In the current culture of medicine, errors will remain a major threat to our patients and our health care system. Errors will also remain a strain on the doctors themselves, which was confirmed by the level of psychological distress that affected our participants. Doctors are known as the second victims of errors because errors can severely impact on their quality of life and on their emotional well-being in particular [5,9,13,22]. Errors also decrease confidence and may leave psychological scars [9,22].

Psychological impact of errors certainly worsens when sexism, as reported by two of our participants, complicates an already painful experience. This created for those female residents an especially *toxic* environment where sexism added to an insufficient culture of safety and error. The finding of perceived sexism in relation to errors aligns with other evidence supporting the persistence of gender-based discrimination in medicine [23-25].

Regarding gender-based differences in experiencing errors, several participants perceived they were more sensitive and probably more affected than their male counterparts. Our participants held these views, although they were aware of their own possible stereotypes. In fact, these findings matched the results of the biggest survey on medical errors (number of participants = 3’171), in which female physicians were almost twice as likely to report stress after an error [9].

Coping - the object of our second research question - is defined as the process to manage stressful demands exceeding one’s own current resources [12]. It is a key point if we think about the distress reported by our participants. Two themes related to coping emerged in our interviews. First, there was the need for our residents to *talk about the errors*. Second, there were the *type of attitudes displayed after an error*, attitudes that can be considered as either constructive or defensive [2]. Both themes - the need to talk and the type of attitudes - can be viewed in the light of Lazarus and Folkman’s work on coping, although these researchers were not focusing on the specifics of medical errors [14].

The need to talk about the error matches the general coping strategy of seeking social support, which is defined as a resource available to an individual in a social environment [14]. Social support can take different forms: it can be emotional support (e.g. reassurance) or informational support (e.g. obtaining advice, feedback). Talking *to the patient* and disclosing the error is likely to have an additional dimension than emotional or informational ones. To describe what happens during error disclosure, some have suggested the religious metaphor of obtaining absolution from the patient [26,27].

Our second theme on coping relates to the types of attitudes displayed after an error. Constructive attitudes in our participants’ accounts belong to an overarching category of coping mechanisms called *problem-focused* by Lazarus and Folkman [14]. In *problem-focused* approaches, the person copes with altering something: environment and/or self. This means altering something to avoid that the error is repeated. An example of altering self would be increased vigilance towards a given clinical picture.

Finally, defensive attitudes in our participants’ accounts belong to the overarching category of coping mechanisms called *emotion-focused*[14]. In *emotion-focused* approaches, the person copes with altering one’s own emotional response to the situation. In defensive attitudes to errors, emotional response is acted upon by simply changing the meaning given to what has happened and thereby reducing one’s own responsibility. As such, defensive attitudes may entail some form of cognitive distortion. Our findings on defensive attitudes align with those of Mizrahi [6]. In his extensive ethnographic study on residents, Mizrahi identified three main defensive attitudes: *denying* that an error occurred, *discounting* part of one’s own responsibility while looking for and blaming any scapegoat, and finally *distancing* to preserve oneself when other defenses do not work (e.g. “Everyone makes mistakes”).

The female residents in our study used coping mechanisms that did not differ from those found in qualitative studies mixing female and male residents [1,3,4,6]. Nevertheless, a gender difference may still exist if we consider the relative use of the various coping mechanisms. This was suggested in Wu’s study showing that female house officers were more likely to make constructive changes after an error than their male counterparts [2]. Outside the specific field of medical errors, gender differences in coping behavior have been found [28-30]. Seeking social support in particular is more common in women’s coping approaches than in men’s ones.

### Strengths and limitations

Although previous qualitative studies *did* explore residents’ experiences with errors [1,3,4,6], our study specifically portrays how female residents in general internal medicine experience medical errors and cope with them. This is an original contribution since such in-depth data are scarce in the literature. These data help us better understand how doctors’ individual profile and characteristics, like gender, can impact on the experience with errors. However, our study clearly lacks explanatory power since our design bears the inherent limitation of not directly comparing female residents with male ones. Only data obtained from both genders would allow such a comparison.

### Area for further research

An area for further research relates to other individual characteristics than gender. How may individual characteristics, such as personality traits, impact on the experience of errors? For example, under relatively similar circumstances (i.e. residency in general internal medicine), only one resident in our study disclosed the error to the patient, and considered it as an effective coping mechanism. For the seven remaining participants, barriers to disclosure appeared difficult to overcome. In the mostly North American literature on medical errors, one of the strongest barriers to disclosure is fear of litigation [8,31]. However, in our local context, litigations are not as big as a concern for doctors. Therefore, we have to better understand how to promote frank disclosures of errors to the patients.

## Conclusions

After having experienced a medical error, the female residents in our study report a high level of emotional distress. This takes place within a professional culture that has a long way to go before errors can be addressed openly and constructively. In addition, gender-based discrimination can occur, thereby worsening this already painful experience. Emotional distress calls for coping mechanisms, among which some result in either constructive or defensives attitudes towards errors. Constructive attitudes mean bringing changes to avoid that the error is repeated whereas defensives attitudes consist of mitigating one’s own responsibility. It is possible that gender differences exist in the relative use of coping mechanisms and therefore in terms of attitudes displayed after an error. Further research allowing for a direct comparison between female and male residents is needed.

## Endnotes

^a^The terms *constructive* and *defensive* are borrowed from previous research on medical errors (e.g. Wu [2]).

## Competing interests

The authors declare that they have no competing interests.

## Authors’ contributions

COM contributed to the study conception. In particular, she introduced the idea of exploring the gender issue within the wider context of medical errors. She conducted all the interviews and was in charge of analyzing the transcripts. She drafted the first version of the paper. GW teaches about medical errors at the University of Lausanne School of Medicine. Hence, he was tasked with commenting and improving the study conception and design. He also critically reviewed the drafts of the paper. DG first conceptualized the research project. He reviewed the whole data analysis and interpretation process. He was in charge of the final version of the paper. All three authors approved the final version of the manuscript.

## Pre-publication history

The pre-publication history for this paper can be accessed here:

http://www.biomedcentral.com/1472-6920/14/140/prepub
